# Computerized handwriting evaluation and statistical reports for children in the age of primary school

**DOI:** 10.1038/s41598-022-19913-y

**Published:** 2022-09-19

**Authors:** Shao-Hsia Chang, Nan-Ying Yu

**Affiliations:** 1 Department of Occupational Therapy, I-Shou University, Kaohsiung City, Taiwan; 2 Department of Physical Therapy, I-Shou University, No. 8, Yida Rd., Jiaosu Village Yanchao District, Kaohsiung City, 82445 Taiwan

**Keywords:** Diagnostic markers, Data integration, Data processing

## Abstract

This study proposed a novel computational method for evaluating logographic handwriting. It can precisely evaluate both the handwriting product and the process. The measures included handwriting performance as well as the temporospatial, kinematics, and kinetics features. For examining the psychometrics of this comprehensive evaluation system, typical development children aged 6 to 9 years old (grade 1 to grade 3) (n = 641) were involved in the study of factor analysis. From twelve measuring variables, the exploratory factor analysis extracted five factors (handwriting performance, motor control, speed and automation, halt and exertion, and “in air” events). The test reliability was confirmed by further recruitment of typically developing children (n = 242). The internal consistency mostly demonstrated good to excellent results for every measure. This study further recruited children with handwriting difficulties (n = 33) for testing the discriminative validity of the evaluation system. A series of two-way ANOVA tests was conducted to test the significance of the main effects of the groups (typical development and handwriting deficit) and grades (1, 2, and 3) and their interaction effects on the handwriting measures. All the measures showed significant differences between the two groups, indicating the discriminative validity for identifying handwriting deficits. Seven of twelve measures showed significant interaction effects, indicating the different trends across the grades between the two groups. Typically-developing children demonstrated ongoing progress from grade 1 to grade 3, suggesting a developmental trend during their early school age. Implications for motor development and clinical evaluation are discussed herein in relation to the five dimensions.

## Introduction

Many researchers argued that handwriting had become a past art since a significant number of digital devices are used in daily life. However, it might be true only in the adult world. For children of primary school, it is impossible for an academic learning process without handwriting. Handwriting is indeed an essential fine motor skill in school-age children. In children with handwriting problems, the learning in classes may become difficult and may lead to low academic performance^1,2^. Such frustration is likely to further lead to their perception of insufficient handwriting skills, consequently they are reluctant to the writing work, and the negative emotions may affect the development of their writing ability. The scrawls of schoolchildren may impact their marks evaluated by teachers. Therefore, handwriting skill is related not only to language learning but affects the development of the self-concept of students^3^.

Handwriting is a complicated process of fine motor movements, which allows for parallel activation of cognitive and motor processes^4^. Poor fine motor control may lead to strenuous or even illegible handwriting. Assessment of fine motor control in handwriting movement is thus important in any comprehensive evaluation of handwriting dysfunction. However, it is difficult to evaluate or analyze the subtle handwriting movements by traditional clinical observation. With the help of a digital tablet, the computerized evaluation provides a quantitative method for measuring specific fine motor control variables during the actual handwriting process^5–10^. For measuring handwriting proficiency, it has been used to document the frequency or duration of pauses in the stroke movement. The poor handwriters can be identified by the increased frequency of pauses or longer pause time^11–13^. Using the Minnesota Handwriting Assessment (MHA) test sheet on a digital tablet, Dirlikov et al. revealed standard (manual derived) MHA and comparable automated handwriting scores were highly correlated. They found elevated letter-form scores (worse performance) in children with ASD and ADHD groups compared to TD children. Children with ASD showed the greatest letter-form impairment^10^. Li‐Tsang et al. (2018) successfully used computerized assessment of temporospatial features and kinetics to distinguish comorbid learning difficulties from attention deficit hyperactivity disorder in Chinese adolescents. They found ADHD and ADHD-LD groups also showed larger variations in either handwriting speed or pen pressure than their controls^14^.

In addition to pauses of stroke movement, the time spent holding the writing instrument above the paper was thought to correspond to the time needed to initiate the handwriting movement to execute the character^9^. The time and trajectory length of ‘‘in air’’ movements have been used to characterize handwriting deficit in fine motor control. As compared with the typically developed group, the non-proficient handwriters showed significantly longer ‘‘in air’’ time and path length^13,15^.

From the aspect of motor control theory, Plamondon defined rapid human movements (automation) in an open-loop paradigm showing a smooth velocity curve with ideally a single peak value^11^. In one of our previous studies, the average number of velocity peaks of a stroke has been applied to characterize the handwriting deficit of children with developmental coordination disorder (DCD). The study showed promising results for using this parameter to characterize motor control problems in children with handwriting difficulties^16^.

Our previous study compared the handwriting process in children with non-proficient characteristics to those of typically-developing children in Grades 1 and 2. We found the attainment of automated handwriting was markedly slower in children with handwriting deficits. The number of velocity peaks was found a valid parameter to monitor the automation of stroke movement in handwriting. The study also revealed the poor handwriters used a faster stroke velocity than typically-developing group to write simple characters but not complex characters, parameters of paramount significance in graphomotor function were adopted to evaluate the children with non-proficient characteristics^16^.

In the recent decade, the advance of artificial intelligence has been implemented on handwriting evaluation for the identification of poor handwriting^17,18^. Wu et al. found that the Support Vector Machine model had the best performance in detecting Chinese handwriting deficit^18^. A total of 34 handwriting features were extracted from the data recorded, which can be generally divided into five categories: spatial, temporal, velocity, acceleration, and pen tilt. Gargot et al. performed K-means clustering to define a new classification of dysgraphia. Twelve digital features describing handwriting through different aspects (static, kinematic, pressure, and tilt) were extracted and used to create linear models to investigate handwriting acquisition throughout education. They found that three features (two kinematic and one static) showed a significant association to predict the change of handwriting quality in control children. In the two clusters exhibiting severe dysgraphia, one presented abnormality in terms of kinematics and pressure whilst the other one showed mainly pen tilt problems^17^. These studies gained new insights into which handwriting features are predictive of handwriting deficits in children and propose a method that can help the clinical and educational professionals to automatically detect children at risk of handwriting deficit. To further the implication of these studies, it is necessary to relate these features to that clinical intervention or educational ecology in handwriting lessons.

Different from the alphabetical language system, a Chinese character is a kind of logogram (or logograph) which means a written character that represents a word or morpheme. In this logographic system, there are many hieroglyphic and cuneiform characters. The difference between the oriental and western language systems should be considered in the design of the evaluation method. There are more than 4000 distinct characters needed for the basic components to acquire the written language skills. The sophisticated character structure forms the major problems in Chinese handwriting. The complicated process of Chinese handwriting has become a complex motor learning and motor control paradigm.

There were few studies or evaluations on legibility, especially in Chinese handwriting that the requirement of legible handwriting is the correct placement of distinct strokes. Different from the alphabetical system, every Chinese character has usually more than ten strokes in a character. The important component of Chinese handwriting is the correct stroke placement with a certain length, the direction of the turn of the strokes in the handwriting. For handwriting accuracy and legibility evaluation, most evaluations of handwriting legibility still relied on eye-ball check. Few studies implemented computer applications for the automated assessment of legibility and accuracy^19–21^. In the work of Li-Tsang et al., they proposed an assessment for calculating accuracy and the total number of characters with stroke errors^21^. However, it is not available how they get the accuracy or legibility data pertaining to the number of characters with added strokes, omitted strokes or concatenated strokes, mirrored strokes and crossing-over strokes. Based on offline image processing, our preliminary system proposed a template matching method for the assessment of legibility, alignment, and size control of Chinese handwriting^19^. For the accuracy evaluation of Chinese handwriting, the work of Hu et al. (2009) was originally aiming at handwriting tutoring. They have proposed a method being able to automatically check handwriting errors. In interactive handwriting learning, attributed relational graph matching is used to locate the handwriting errors, such as the stroke production errors, stroke sequence errors, and stroke relationship errors^20^. All the results of the above studies showed the relevance of combining error detection and legibility assessment of individual strokes with the traditional kinetic and kinematic handwriting evaluation. A more scientific definition with clinical meaning and the computing of legibility and accuracy are needed in a comprehensive evaluation.

In a classroom setting, school teachers need a standardized practical tool for the measurement of legibility to help them identify those with difficulties and determine whether the child needs support to develop their skills^22^. In the clinical setting, therapists need an evaluation of the issues related to motor learning and motor control aspects. The present system has addressed legibility analysis and the temporospatial features of the handwriting process. This paper proposed a novel computational method for the evaluation of logographic handwriting. It can precisely evaluate both the handwriting product and the process. The aim of the current study was therefore to develop a reliable and valid computerized assessment of the temporospatial features and performance (speed and legibility) of handwriting. The discriminative validity was examined by the comparison between children with typical development and handwriting difficulties. For constructing a reference for clinical implementation, the differences were also examined between genders and across the school years.

## Methods

### Participants

In this study, 655 children aged 6–9 from elementary school were recruited to participate in the experiments for verifying the construct of evaluation and the reliability of the testing measures. After excluding children with neuromotor or related medical diagnoses (such as cerebral palsy, muscle atrophy, developmental retardation, and intellectual disability), a total of 641 children were chosen. As eight/six children were diagnosed with developmental retardation disorder, their data have been excluded from the statistical analysis. The final subject number in factor analysis was 342 girls and 299 boys.

A second sample from another 242 children (129 girls and 113 boys) was collected for the reliability test and statistical analysis to construct a reference for future clinical implementation of this evaluation system.

This study further recruited children with handwriting difficulties to check whether the tests can discriminate against them from children with typical development. The handwriting deficit has been confirmed by the administration of the Chinese Handwriting Evaluation Form (CHEF)^23^. The CHEF has 29 items that measure six handwriting dimensions, including the construction of characters, major mistakes in writing the components of characters, developmental delays, use of a pencil, a gross motor function, and emotional reaction to the problem. According to the test manual, the cut-off criterion for the identification of a handwriting deficit is the presence of difficulties in two or more of the six dimensions, with a median score larger than, or equal to three^23^. Of 395 children, thirty-three children with handwriting difficulties were identified and then recruited into the study for the validity analysis of the tests.

All participants and their parents gave written informed consents. The Institutional Review Board of the E-DA Hospital approved the study (EDAH IRB No. EMRP14107N). All participants and their parents gave written informed consents. All the consents were documented and witnessed by the Institutional Review Board of the E-DA Hospital. Table 1 shows the basic information and descriptive statistics of the participants recruited for reliability and discriminative validity tests.

**Table 1:** Basic information and descriptive statistics of participants recruited for reliability and discriminative validity tests.

Grade	Participant number	Age mean (STD)	Boy/girl number (proportion)
**TD**			
1	103	6.34 (0.18)	48 (46.7%)/55 (53.3%)
2	73	7.04 (0.21)	34 (46.6%)/39 (53.4%)
3	66	8.11 (0.20)	31 (47.0%)/35 (53.0%)
**HWD**			
1	13	6.38 (0.24)	7 (53.8%)/6 (46.2%)
2	10	7.22 (0.23)	8 (61.5%)/5 (38.5%)
3	10	8.10 (0.15)	6 (60.0%)/4 (40.0%)

*TD* Typically developing children, *HWD* Children with handwriting difficulties.

### Instruments and apparatus

Participants were seated on a chair in front of a desk on which a digitizer tablet (487 × 318 × 12 mm, PH-1820-A, PendoTech, ShangHai) was positioned so that the tablet’s lower edge lined up with the edge of the table at which the participant was seated. On the digitizer tablet, an A4-sized piece of paper was positioned with the vertical and horizontal edges parallel to the horizontal and vertical edges of the digitizer. A standard-sized wireless electronic inking pen with a force-sensitive tip (2048 levels) was used to collect the movement data on the digitizer tablet. The axial pen force and X (horizontal) and Y (vertical) positions of the pen tip were sampled at a frequency of 200 Hz with a spatial resolution of 0.01 mm. This data acquisition setup was adopted from our previous study for neurodegenerative disease with the exception of digital tablets used^24^.

### Copying target

**Figure 1:**
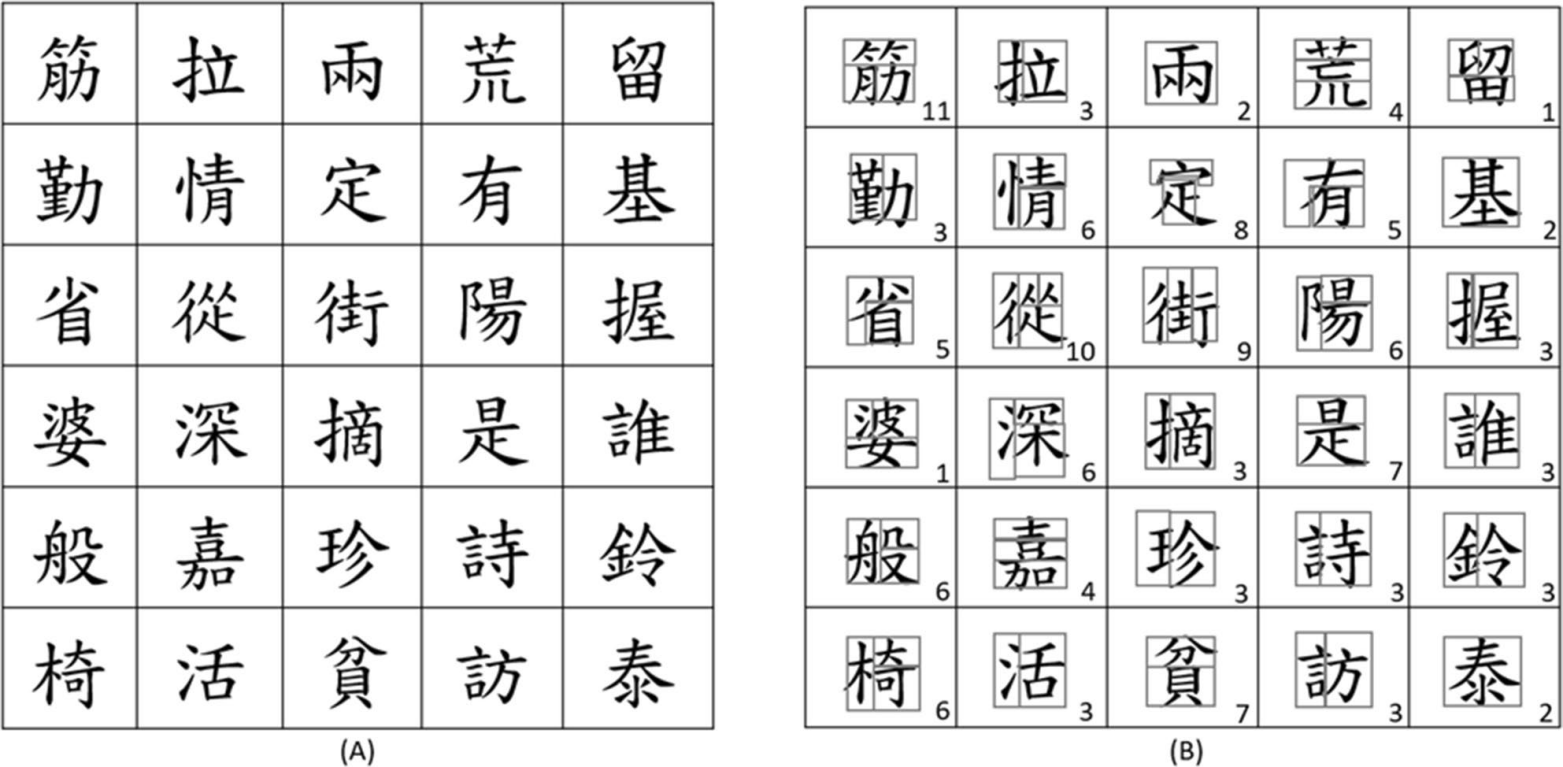
(**A**) The copying targets of Chinese characters. (**B**) The configuration of characters with segmented components.

Figure 1A shows the characters used in this study as copying targets. According to the stroke number of most used Chinese characters, thirty Chinese characters were selected and printed on the gridded A4-size white paper. The characters were selected from the Chinese textbooks for schoolchildren in Grades 1 and 2 to ensure that the children have learned the characters. Figure 1B shows the configuration of the components in every Chinese character. According to the configuration, every character can be classified into one of the eleven categories in the copying target. The number shown on the corner of the grid denotes their attribute of the categories. During the test, the writing space was blank grids with the same format as shown in Fig. 1A.

### Measuring parameters

The measures of computerized handwriting evaluation.

#### Performance measures

**Figure 2:**
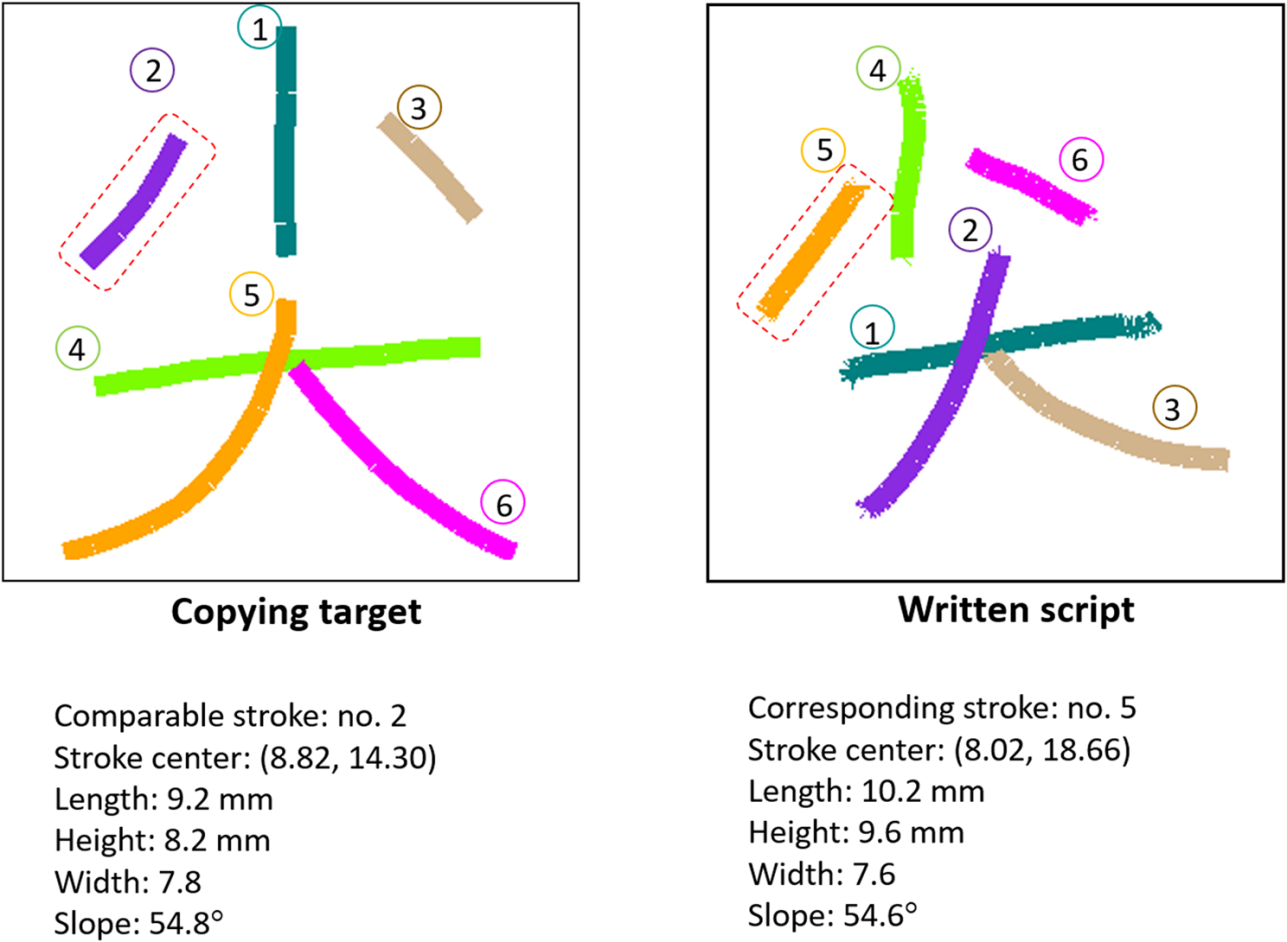
The comparison between copying target and written script. Given as an example, the parameters of the second stroke of the copying target are compared with the corresponding stroke of the written script (no. 5).

1. Writing speed (WS) According to the number of characters written in ten minutes, the speed is computed then expressed as characters per minute. If the time to complete all the tasks is less than ten minutes, the test time is modified according to the time they really took.
2. Correct writing speed (CWS): Correct writing of Chinese characters is defined as all the strokes are correctly placed with their corresponding positions, lengths, widths, heights, and directions. Following these rules, the evaluation system defined the criteria of correctness that all strokes being correctly placed as demonstrated on the left of Fig. 2. A sample of twenty-two test sheets including 566 characters was used for the validation of this categorization with an eye-ball check. High agreement (Kappa = 0.92, p < 0.001) was found between automatic check and eye-ball check by an experienced elementary school teacher. According to the number of characters written correctly in ten minutes, the correct writing speed is computed and then expressed as the accurate number of characters written per minute.

#### Geometric measures (legibility)

1. *Intra-character configuration (ICC)* This measures the ability to control stroke placement within a character. A Chinese character is composed of strokes up to tens or twenties, and even more than thirty strokes. In legible handwriting, every stroke should be appropriately placed to correctly express the configuration of the character. For every stroke, five measures were used to evaluate the appropriateness of stroke placement. The evaluation of intra-character configuration includes the measures of location, width, height, length, and slope of the stroke. Figure 2 shows an example of a comparison between the target and writing scripts. Given the second stroke of the target task and the fifth stroke of writing script as a comparable example, the differences in the five measures are computed to show the deviation from standard character configuration. The layout of a standard character is in a square grid (20 × 20 mm). Every written character was rescaled to fit in the square grid. The maximum differences between standard and handwritten scripts are 28.28 mm in a central location and stroke length, 20 mm in width and height, and 90 degrees in the slope. With a normalization to the maximal values, the proportion of differences would be deducted from the ideal score of 100 (%), then the average value of the total 30 characters was calculated.
2. *Character size (CS)* Character size is defined by the area of a virtual grid that the written character can exactly fit. The area is the product of the character width and height. The unit of this measure is mm^2^.
3. *Character Size Consistency (CSC)* The standard deviation of the character size (area) is computed to indicate the ability to control a character with the same size. The unit of this measure is mm^2^.

#### Temporospatial measures

1. *Accuracy of Stroke order (ASO)* Every Chinese character has its habitual order to write down the strokes. There are general rules of stroke order that have been defined and taught by the education system of countries with Chinese as their official language. Coming from ergonomic analysis of the context in Chinese handwriting, the general rule of stroke order includes (1) from top to bottom, (2) from left to right, and (3) from outside to inside. Using the metrics of mean absolute error (MAE)^25^, the accuracy of stroke order was calculated by subtracting the stroke order of the sample characters from the stroke order of the written script, and the absolute value represents the error degree. For example, in writing “尖”, the proper sequence (denoted by number on Fig. 2) should be the upper component (3 strokes: middle → left → right) then the lower one (3 strokes: horizontal → center to left bottom → center to right bottom). When children write the lower component first, the fourth stroke they write is the first stroke in the correct order, so the error value of the first stroke was |4–1| = 3, the second stroke was also 3 (|5–2|) and so on. If two strokes were completed by one stroke, the second stroke of the order would be accumulated as an error value. For example, the xth and (x + 1)th strokes were written as a single stroke. The (x + 1)th stroke accumulates an error value |(x + 1) − x|. The same as in one stroke was written as two distinct strokes. An error value was accumulated |(x + 1) − x|. The completely reverse stroke order was taken as the maximum value of the wrong stroke order. The sum was calculated by subtracting the maximum value with the absolute value of error degree, the proportion of this value to the maximum value of wrong stroke order represented the proportion of correct stroke order, which was presented in percentage.
2. *Pen pause time ratio (PPTR)* This measures the dysfluency of movement from the temporal data. If two consecutive sampling points had the same registered coordinate, the period was cumulated and referred to as pause time. This parameter is derived by dividing the cumulative pause time by the total stroke number.
3. *On-paper time ratio (OPTR)* This parameter measures the temporal proportion of the pen-tip contacts on paper. It is derived by dividing the total on paper time spent on the task by the total time including the halting time in the air.
4. *On-paper length ratio (OPLR)* This parameter measures the proportion of stroke trajectory length on the paper. It is derived by dividing the total on paper trajectory length by the total pen tip trajectory length including the wandering length in the air.
5. *The number of velocity peaks per stroke (NVP)* This measure is one of the kinematic features. The number of vertical (or horizontal) velocity peaks for every vertical (or horizontal) stroke was determined as quantification of automation of handwriting movement. The NVP per stroke decreases as the movement becomes automatic; otherwise, the movement remains deliberate. When movement is fully automated, the ideal NVP per stroke is 1 per vertical or horizontal stroke; a decrease in the number indicates a switch from closed feedback control to open-loop control^7,26^.
6. *Mean peak velocity (MPV)* This measures one of the kinematic features. The maximum value of the tangential velocity per stroke was first determined, after which the mean peak velocity per trial was determined by averaging the values across the strokes. The mean peak velocity per condition was determined for every participant by averaging the values of all trials per condition.
7. *Axial pen force (APF)* This measures the kinetic feature. Since force recorded at the start and end of a stroke has large variation, it cannot represent the valid exerted force. For avoiding the effect of this variation, the axial pen force only in the middle 80% of a stroke (ex. middle 4 mm of a 5-mm stroke) was recorded^16^. The mean value was determined by dividing the sum of these values by the total number of sampling points in a task.

### Data analyses

The collected data were loaded into the computer for further analysis. SPSS for Windows EC (Version 18.0) was applied in the analysis of these data. The test data were analyzed by exploratory factor analysis. Principal component analysis (PCA) with varimax orthogonal rotation was used to explore the main dimensions these 12 measures converge. For realizing the internal struct of the measure items, Pearson correlation analyses were conducted to examine the intercorrelation among the twelve measures.

### Reliability tests

We examined the consistency of the measurement variables using Cronbach’s α^27^. Cronbach’s α was calculated as an index of the internal reliability of the measurement system. To test the measuring results' consistency, every copy test trial (copying 30 characters) was split into three sections. Every section contained the process of copying 10 characters. A reliability coefficient across the three sections was obtained for each variable from the 242 typically developing children. High inter-trial coefficients would indicate that the trials are measuring the same underlying construct.

### Statistical analyses

For the discriminative validity of the evaluation system, a series of two-way analysis of variance (ANOVA) tests were conducted to test the significance of main effects of the groups (typical development and handwriting deficit) and grades (1, 2, and 3) and their interaction effect on the handwriting measures. A series of independent t-tests were also performed to compare the measures between two genders. The statistical significance level is set at 0.05.

### Ethical approval

All procedures performed in studies involving human participants were in accordance with the ethical standards of the institutional and/or national research committee and with the 1964 Helsinki declaration and its later amendments or comparable ethical standards. The Institutional Review Board of the E-DA Hospital approved the study (EDAH IRB No. EMRP14107N).

### Informed consent

All participants and their parents gave written informed consents. All the consents were documented and witnessed by the Institutional Review Board of the E-DA Hospital. All possible harms or unintended effects in each group were depicted in the consent.

## Results

### Factor analysis

Factor analyses were conducted on the 12 test measures. With a large sample size (N = 611) and a high subjects-to-variables ratio (53.4), it is acceptable in predicting important outcomes in exploratory factor analysis^27^. The KMO value is 0.528 (> 0.5). It supports the sampling adequacy for factorability as suggested by Kaiser^28^. Bartlett's test of sphericity reaches statistical significance (p < 0.001). It indicates the correlation matrix is not an identity and supports the factor analysis^29^. After varimax orthogonal rotation, five major components with eigenvalues larger than 1 were extracted (Table 2). From the largest to the smallest, the eigenvalues were 3.303, 2.036, 1.831, 1.432, and 1.158. Factor loadings larger than 0.5 were observed. The five factors accounted for 81.33% of the total variance. The first dimension contains 3 measures (Nos. 2, 3, 6) relevant to handwriting performance (correct writing speed, legibility, and accuracy of stroke order). The second dimension contains the measures (Nos. 4 and 5) relevant to the character size control and the average of peak velocity (No. 11). It is related to motor control in handwriting. The third dimension contains two measures relevant to the speed to complete writing characters (No. 1 and 2) and the movement automation (No. 10) in stroke production. The fourth dimension contains the time measure of the pen halting on paper (No. 7) and the axial pen tip force (No. 12). The fifth dimension contains two measures (Nos. 8–9) relevant to the proportion of time spent and trajectory length really on paper.

**Table 2:** Factor analysis: measures and factor loadings (N = 641).

	Factor				
	1	2	3	4	5
1. Writing speed			0.785		
2. Correct writing speed	0.712		0.524		
3. Stroke placement control	0.943				
4. Character size		0.861			
5. Character size consistency		0.873			
6. Accuracy of stroke order	0.927				
7. Pen pause time ratio				0.861	
8. On-paper time ratio					0.912
9. On-paper length ratio					0.754
10. No. of velocity peaks per stroke			− 0.909		
11. Mean peak velocity		0.592			
12. Axial pen force				0.792	
Eigenvalue	3.303	2.036	1.831	1.432	1.158
% variance	20.205	15.943	15.734	14.946	14.506
Cumulative % variance	20.205	36.149	51.882	66.827	81.333

Measures with factor loadings larger than 0.50 are listed. % is the variation explained by a single factor. Cumulative % is the variation explained cumulatively.

Table 3 shows the intercorrelation among the testing measures. Some of the measures were found to correlate with others indicating the interrelated metrics. The measures of writing speed (nos. 1 and 2) were found to significantly correlate with all the others except for character size consistency. The measure in the proportion of trajectory length on paper was also found to significantly correlate with all others except the intra-character configuration. With the least other measures, the character size consistency was found to correlate with only five other measures. A similar result in the intra-character configuration was also found to correlate with six other measures.

**Table 3:** Cross correlation among the computerized measures (N = 641).

	WS	CWS	ICC	CS	CSC	ASO	PPTR	OPTR	OPLR	NVP	MPV	APF
WS		0.719***	0.158***	− 0.204***	0.035	0.370***	− 0.216***	0.305***	0.456***	− 0.551***	0.411***	0.131***
CWS	0.000		0.595***	− 0.144***	0.018	0.692***	− 0.168***	0.201***	0.318***	− 0.412***	0.285***	0.090*
ICC	0.000	0.000		0.027	− 0.158***	0.850***	0.051	0.049	0.047	− 0.091*	0.044	− 0.155**
CS	0.000	0.000	0.496		0.689***	− 0.091*	0.202***	− 0.003	0.108**	0.120**	0.279***	− 0.178***
CSC	0.187	0.338	0.000	0.000		− 0.142***	− 0.044	− 0.031	0.128**	0.046	0.321***	− 0.023
ASO	0.000	0.000	0.000	0.021	0.000		− 0.039	0.034	0.127**	− 0.251***	0.135***	− 0.082*
PPTR	0.000	0.000	0.097	0.000	0.270	0.159		0.240***	− 0.117**	0.028	− 0.207***	− 0.481***
OPTR	0.000	0.000	0.107	0.936	0.436	0.195	0.000		0.484***	0.130***	− 0.041	0.082*
OPLR	0.000	0.000	0.116	0.006	0.001	0.001	0.001	0.000		− 0.188***	0.268***	0.466***
NVP	0.000	0.000	0.010	0.002	0.240	0.000	0.234	0.000	0.000		0.061	0.050
MPV	0.000	0.000	0.133	0.006	0.000	0.000	0.000	0.150	0.000	0.060		0.119**
APF	0.000	0.011	0.002	0.000	0.562	0.018	0.000	0.018	0.000	0.103	0.001	

*WS* Writing speed, *CWS* Correct writing speed, *ICC* Intra-character configuration, *CS* Character size, *CSC* Character size consistency, *ASO* Accuracy of stroke order, *PPTR* Pen pause time ratio, *OPTR* On-paper time ratio, *OPLR* On-paper length ratio, *NVP* Number of velocity peak per stroke, *MPV* Mean peak velocity, *APF* Axial pen force.

*p < 0.05, **p < 0.01, ***p < 0.001.

### Instrument reliability

Table 4 shows the results of the instrument reliability testing where each of the replications is considered an independent test. All of the computerized handwriting measures exhibited high repeatability (with Cronbach’s α coefficients ranging from 0.751 to 0.968). The relatively low coefficient for the On-paper length ratio (0.751) indicates that different parts of the copying target with different complexities may exhibit varied trajectory lengths of pen movement in the air.

**Table 4:** Reliability estimates across trial replications for the evaluation variables for 242 children.

	Cronbach’s α
Writing speed	0.900
Correct writing speed	0.895
Stroke placement control	0.854
Character size	0.803
Character size consistency	0.854
Accuracy of stroke order	0.891
Pen pause time ratio	0.832
On- paper time ratio	0.832
On-paper length ratio	0.751
Number of velocity peak per stroke	0.929
Mean peak velocity	0.968
Axial pen force	0.968

### Discriminative validity

﻿Table 5 shows the result of comparisons between children with typical development and those with handwriting difficulties among the three grades. All measures show significant differences between the two groups indicating the discriminative validity for identifying handwriting deficits. All measures except mean peak velocity showed significant main effects from the grade factor. Seven measures showed significant interaction effects indicating the different trends across the grades between TD and HWD groups. Further post hoc tests on the TD group found significant differences between adjacent grades in the measures pertaining to performance, intra- and inter-character configuration (legibility), and the temporospatial variables. The other six measures did not display significant differences between grade 2 and grade 3. It includes the measures of stroke placement, stroke order accuracy, and the temporospatial features related to movement automation and motor control. However, all these measures display the ongoing progress of these skills from grade 1 through grade 3 in children with typical development.

**Table 5:** The comparison of testing measures among three grades and two groups.

		WS	CWS	ICC	CS	CSC	ASO	PPTR	OPTR	OPLR	NVP	MPV	APF
**TD**													
Grade 1 N = 103		3.03 (1.1)	1.88 (0.51)	77.21 (6.21)	185.64 (41.37)	43.94 (10.85)	72.20 (11.29)	2.34 (1.77)	30.29 (6.90)	25.16 (4.70)	3.08 (1.08)	4.14 (1.89)	77.97 (13.22)
Grade 2 N = 73		5.42 (1.31)	3.82 (1.01)	80.26 (4.60)	153.01 (47.32)	34.68 (11.86)	71.02 (9.42)	0.78 (0.76)	33.54 (6.82)	29.14 (4.06)	2.35 (1.34)	5.01 (2.66)	82.85 (10.93)
Grade 3 N = 66		8.22 (1.72)	5.90 (1.38)	80.70 (5.60)	123.64 (32.45)	27.28 (7.15)	83.62 (11.00)	0.41 (0.56)	38.00 (5.59)	31.09 (4.27)	1.93 (1.15)	5.28 (2.55)	85.86 (8.24)
**HWD**													
Grade 1 N = 13		1.09 (0.33)	0.87 (0.78)	51.72 (12.71)	218.83 (37.01)	68.00 (12.34)	41.07 (18.55)	3.66 (1.03)	23.33 (4.41)	22.16 (4.27)	7.41 (5.18)	0.78 (0.22)	65.25 (23.04)
Grade 2 N = 13		1.21 (1.23)	0.53 (0.47)	54.46 (13.84)	214.86 (53.47)	55.62 (10.04)	48.09 (18.95)	2.46 (0.72)	28.44 (3.42)	25.93 (4.13)	5.93 (3.95)	0.56 (0.46)	40.58 (19.92)
Grade 3 N = 10		2.41 (1.70)	1.06 (0.75)	64.69 (12.93)	211.68 (51.35)	53.80 (6.11)	42.94 (22.47)	2.99 (0.81)	24.96 (5.43)	27.44 (5.33)	4.21 (2.20)	0.70 (0.27)	56.17 (28.00)
*F* (Group)		276.3	243.5	965.9	65.5	105.62	269.5	70.8	54.0	17.2	113.9	110.2	138.3
*P* value		0.000	0.000	0.000	0.000	0.000	0.000	0.000	0.000	0.000	0.000	0.000	0.000
*F* (Grade)		59.4	24.8	16.2	6.86	15.08	5.5	17.5	7.1	17.6	15.2	0.6	7.5
*P* value		0.000	0.000	0.000	0.001	0.000	0.005	0.000	0.001	0.000	0.000	0.540	0.001
*F* (Interaction)		77.5	21.3	12.7	4.31	0.24	1.3	2.7	4.1	0.05	3.3	1.0	14.0
*P* value		0.000	0.000	0.000	0.014	0.786	0.268	0.071	0.018	0.947	0.037	0.369	0.000
Post hoc test^#^		3 > 2 > 1	3 > 2 > 1	3 > 1 2 > 1	1 > 2 > 3	1 > 2 > 3	3 > 1 2 > 1	3 > 1 2 > 1	3 > 2 > 1	3 > 2 > 1	3 > 1 2 > 1	3 > 1 2 > 1	3 > 1 2 > 1

*TD* Typical development, *HWD* Handwriting deficit, *WS* Writing speed, *CWS* Correct writing speed, *ICC* Intra-character configuration, *CS* Character size, *CSC* Character size consistency, *ASO* Accuracy of stroke order, *PPTR* Pen pause time ratio, *OPTR* On-paper time ratio, *OPLR* On-paper length ratio, *NVP* Number of velocity peak per stroke, *MPV* Mean peak velocity, *APF* Axial pen force.

^#^The post hoc test shows the comparisons between grades in the TD group.

Table 6 shows the result of comparisons between girls and boys. The girls show better performance in all the measures. Five measures were found to reach statistical significance, it includes correct writing speed, character control, stroke order, and the axial pen force. It includes the measures of stroke placement, stroke order accuracy, and the temporospatial features related to movement automation and motor control.

**Table 6:** The comparison of testing measures between genders.

	WS	CWS	ICC	CS	CSC	ASO	PPTR	OPTR	OPLR	NVP	MPV	APF
Girl N = 129	5.33 (2.58)	3.92 (1.38)	80.53 (4.88)	153.09 (48.53)	34.92 (11.80)	80.52 (10.52)	1.19 (1.38)	33.74 (6.54)	27.65 (5.04)	2.42 (1.11)	4.49 (2.18)	83.25 (10.70)
Boy N = 113	5.10 (2.49)	3.54 (1.10)	78.21 (5.43)	161.65 (46.88)	38.56 (12.57)	76.65 (10.91)	1.45 (1.49)	32.95 (7.91)	28.31 (5.15)	2.64 (1.41)	4.83 (2.42)	79.88 (12.74)
t statistics	0.693	2.294	3.494	− 1.391	− 2.319	2.808	− 1.419	0.805	− 1.005	− 1.307	− 1.170	2.238
p value	0.489	0.023	0.001	0.166	0.021	0.005	0.157	0.396	0.316	0.193	0.243	0.026

*WS* Writing speed, *CWS* Correct writing speed, *ICC* Intra-character configuration, *CS* Character size, *CSC* Character size consistency, *ASO* Accuracy of stroke order, *PPTR* Pen pause time ratio, *OPTR* On-paper time ratio, *OPLR* On-paper length ratio, *NVP* Number of velocity peak per stroke, *MPV* Mean peak velocity, *APF* Axial pen force.

## Discussion

The result of this study confirmed the reliability and validity of the comprehensive computerized evaluation of Chinese handwriting. There are five major dimensions revealed for the evaluation of children from grades 1 to 3. The first three dimensions display the measures of handwriting performance. Dimension 1 includes the measures directly related to academic learning (correctness, legibility, and stroke order). Dimension 2 includes the measures related to motor control (character size control and peak stroke velocity). Dimension 3 is composed of measures displaying the automation of handwriting (copying speed and number of velocity peaks). Dimension 4 shows the interrelation between kinetic and kinematic measures. A greater force applied results in much slower pen tip stroke movement (as reflected by a much greater pause ratio). Dimension 5 is the measure of the on-paper to in-air ratio. It measures the temporal and spatial efficiency of handwriting.

For measuring the general handwriting performance, this study presented descriptive statistics of computerized handwriting measures in school children from grade 1 to grade 3 and the discriminative validity for identifying handwriting deficit. Children with handwriting difficulties showed significant impairments in all measures. In addition to the traditionally kinematic and kinetics results, this study showed the result of structural deficits in children with handwriting difficulties. The strength of this evaluation system is not only the performance deficit can be identified but the process of handwriting can also be provided to clinicians or parents of the children. The general handwriting performance was found significantly improved across the three grades. Gender differences can be found in five measures. Girls showed significantly better performance than boys in all measures of Dimension 1 (CWS, ICC, and ASO) and CSC in Dimension 2. They also showed increased force exerted on the paper (APF) than boys. With reference to the norm of these data, the differences between genders should be considered.

Up to date, pen-and-paper tests and observation scales are the most commonly used handwriting evaluation in school or clinical settings. These evaluations are conducted by eye-ball check which is subjective and difficult to be quantified. For providing quantitative measures, the present computerized system is not only able to count the number of completed characters but also the number of characters being correctly written. This is the most challenging process for a computerized system since there are no standard criteria available even for eye-ball checks. For example, the character “夫” is different from “天” because the middle strokes start from different heights. For correct writing of the later character, what is the minimum height of the starting position is acceptable? In addition, all the geometric relations of any stroke with other ones are related to the judgment of accurate writing. In the present system, the criteria for judgment are the correctness of all strokes in a character.

Since Chinese characters are composed of distinct strokes. Correct writing is defined as all the strokes are correctly placed with their corresponding positions, lengths, widths, heights, and directions. In a previous study for the evaluation of fine motor function, there was a new attempt to score the legibility of Chinese handwriting. It has been used for detecting the early sign of fine motor deficit in persons with mild cognitive impairment^24^. The evaluation tool proposed in this study is a novel implementation on the legibility assessment for children in early school age. From the result of this study, children in grade 2 seem to reach a certain level of the stroke placement control whereas the character size and its variation seem to decrease persistently to grade 3.

To evaluate the motor control of handwriting, the present system includes the assessment specific to movement automation and movement control. Our previous study revealed slower stroke velocities in children with DCD than in those without DCD. The mean peak stroke velocity (MPV) can reflect the fluency of pen movement^16^. Another previous study also found increased pause time per stroke and an increased number of velocity peaks in children with dysgraphia. These two measures (PPTR and NVP) can reflect the automation of stroke formation in clinical handwriting evaluation^13^.

With a standardized process, the pen pause time ratio of pen-tip movement (PPTR) reflects the movement smoothness. Pauses during handwriting have been proposed to be greatly affected by linguistic processes^4^. As revealed from the result of this study, the pen-tip movement in strokes of handwriting becomes more mature as the increase of age. In the sample of typically-developing children in this study, the significant difference was only found between grades 1 and 2 but not 2 and 3. It indicates that the change of this measure may reach a plateau around the age of grade 2.

In the measure of legibility, different from the alphabetical language system, the key point of Chinese character is the spatial relationship of strokes within a character. The tempo and rhythm of stroke placement with an ideal stroke sequence form fluent and legible handwriting. The present system is the first evaluation including stroke order evaluation. This is a particular evaluation that may be only found in the logographic language system. Being included in Dimension 1, this measure shows its relevance in handwriting performance evaluation. As revealed from the results (Table 3), it is found highly correlated with intra-character configuration (r = 0.815). Children with higher ratios of correct stroke sequence exhibited higher scores in intra-character configuration. It implicates an important factor determining Chinese handwriting legibility.

For the progress of handwriting, there are two parameters showing the halt of the progress in the air: the accumulation of “In air” time and the “In air” length. These two parameters show different meanings. The “In air” length indicates the seeking path but the “In air” time may just reflect the halting time. A previous study found that the “In air” time measure may supply information about the perceptual aspect of the motor act^4,30,31^. By comparing with normal controls, the “in air” information in handwriting shows the applicability in the diagnosis of neurodegenerative disease (Parkinson’s and Alzheimer’s disease)^31^. It has been considered to relate to the difficulty with motor memory for letter formation or difficulty in visualizing the letters as needed to form them rapidly^9^. This study reveals these two parameters form the fifth dimension of the evaluation system. They were also found significantly correlated with the other dynamic measures. The significant differences across the three grades indicate the progressive improvement in the early school age (grades 1 to 3).

In the kinetic analysis of handwriting movement, poor writers have been observed to have higher pressure exerted on the writing surface during their writing tasks^6^. However, different results were reported by Rosenblum and Livneh-Zirinski in both the name writing and the paragraph copying tasks, the children with DCD exerted significantly lower mean pressure in comparison to the control children^32^. Our past work also found a dramatic decrease in the axial pen pressure in children with DCD when they were copying complex characters^16^. The present study showed a similar result to our past work and that of Rosenblum and Livneh-Zirinski^32^. Children with handwriting difficulties were found to exert less pressure than typically-developing children. In the typically-developing group, the axial pen force was found to increase with the increase of age but only between grades 1 and 2. Further study is needed to confirm at what age the increase of pen tip pressure stops.

### Suggestions for the application of this system

This study proposed a novel computerized handwriting evaluation system for screening children with handwriting difficulties. Since significant differences between genders were found in correct writing speed, intra-character configuration, character size consistency, the accuracy of stroke order, and axial pen force, the score conversion of these measures are suggested to be different between girls and boys. However, as standardized norm references for future clinical applications, more data on participants such as information about cognitive abilities, socioeconomic status, and other relevant variables should be provided. In the present stage, it can be utilized as a screening for handwriting deficits or as a test to add valuable information during clinical or educational assessments.

## Limitations

Some limitations should be noted. The first is the pen used cannot be their usually used pencil. For this limitation, future study is suggested to develop a system equipped with pencils used in primary school instead of the currently used ballpoint pen.

The second limitation is that the evaluation of characters or paragraph alignment control is not available in the present study. There are grid lines on the test sheet. It may refrain to detect poor alignment control when no grid lines are provided. Further study and the evaluation design may have tasks without grid lines.

## Conclusion

This computerized test provides an on-site realistic evaluation and a quantitative report of the quality of the handwriting product. The focus was on the assessment of a naturalistic (and therefore ecologically valid) handwriting task, which is easy to gather from large numbers of children in the usual classroom setting. Through the computerized analysis, the accuracy and legibility can be scored by the computer instead of the eye-ball check. The development of a computerized evaluation presented in this paper shows the relevance of providing objective assessment results to the related team members for the plan or discussion of an effective intervention. For educators, clinicians and researchers, this evaluation system can provide greater insight into the motor, perceptual and cognitive components underlying poor handwriting. The current results open new opportunities for screening and detection of children with handwriting difficulty in the classroom.

## Data Availability

The datasets used and/or analyzed during the current study are available from the corresponding author on reasonable request.
